# Core mutations, *IL28B *polymorphisms and response to peginterferon/ribavirin treatment in Swedish patients with hepatitis C virus genotype 1 infection

**DOI:** 10.1186/1471-2334-11-124

**Published:** 2011-05-12

**Authors:** Erik Alestig, Birgitta Arnholm, Anders Eilard, Martin Lagging, Staffan Nilsson, Gunnar Norkrans, Thomas Wahlberg, Rune Wejstål, Johan Westin, Magnus Lindh

**Affiliations:** 1 Department of Infection and Virology, University of Gothenburg, Gothenburg, Sweden; 2 Infectious Diseases Clinic, Borås Hospital, Borås, Sweden; 3 Chalmers University of Technology, Department of Mathematical Statistics, Gothenburg, Sweden; 4 Infectious Diseases Clinic, Central Hospital, Skövde, Sweden

## Abstract

**Background:**

Patients infected with hepatitis C virus (HCV) genotype 1 respond poorly to standard treatment with 50% or less achieving sustained virologic response. Predicting outcome is essential and could help avoid unnecessary treatment and reduce health cost. Recently, an association of amino acid substitutions in the core region and treatment outcome was observed in Japanese patients. In the present study, the impact of these mutations on response kinetics and treatment outcome was explored in Caucasian patients.

**Methods:**

The core region of HCV pre-treatment samples obtained from 50 patients treated with peginterferon/ribavirin in a previous Swedish clinical trial with genotype 1 infection were sequenced. The alleles at rs12979860, a single nucleotide polymorphism (SNP), were assessed in order to identify any co-association with this strong response predictor.

**Results:**

No association between treatment response and substitutions of core residue 91 was found. In contrast, substitutions of core residue 70 were observed in 6/21 (29%) non-responders, but only in one of 29 responders (p = 0.03), and were more common in subgenotype 1b (R70Q in 6 of 13 strains) than in 1a (R70P in 1 of 37 strains, p = 0.004). The rs12979860 SNP upstream of the IL28B gene was overall the strongest response predictor (p = 0.0001). Core 70 substitutions were associated with poorer response kinetics in patients carrying the CT genotype at rs12979860.

**Conclusions:**

The results indicate that substitutions of core residue 70 are related to treatment response in Caucasian patients with HCV-1b infection, but are of less importance than IL28B polymorphism.

## Background

Hepatitis C virus (HCV) infection is a major cause of cirrhosis and hepatocellular cancer affecting approximately 170 million persons worldwide [1]. Combination therapy with pegylated interferon and ribavirin (Peg-IFN/RBV), given for 24 to 72 weeks, may eradicate the infection and stop progression of liver damage [2-4], but many patients do not achieve sustained virologic response (SVR). For this reason, and in light of high costs and frequent side effects, it is important to identify factors that can predict the likelihood of response.

Several host factors such as age, stage of liver fibrosis, body mass index (BMI), liver steatosis, insulin resistance and ethnicity, as well as viral genotype influence the treatment outcome [5]. While the impact on outcome by genotype is undisputed, with 80% of patients with genotype 2 or 3 achieving SVR as compared with 50% for genotype 1, the importance of subtypes [6], regional variability or mutations are controversial. In 1995 it was reported from Japan that mutations in a part of the NS5A region were associated with treatment response in genotype 1b patients. The association between mutations in this segment of NS5A, denoted the interferon sensitivity determining region (ISDR) [7] was verified in subsequent Japanese, but not in European studies [8-10].

Several reports have described that Japanese patients carrying HCV strains with a substitution of amino acids 70 (R70Q) and 91 (L91M) respond less well to treatment [11-13], and it was suggested that pre- and on-treatment analysis of core region polymorphism may be useful for predicting SVR in individual patients [14]. However, the occurrence of core 70 and 91 substitutions has not been corroborated in European strains.

In addition to well-known host factors like age and liver fibrosis, it was recently revealed that single nucleotide polymorphisms (SNPs) upstream of the interferon λ3-encoding *IL28B *gene may predict which patients achieve SVR after the completion of peg-IFN/ribavirin therapy [15,16]. This observation may be relevant for investigations of how core variation influences treatment response in different populations of patients with hepatitis C, firstly because the distribution of *IL28B *SNP genotypes differ between ethnic groups [15,17] and secondly because SNP differences may bias the results.

In this study, serum samples were drawn from 50 patients infected with HCV genotype 1a or 1b prior to treatment with peginterferon-alpha2a and ribavirin for sequencing of the core region and part of NS5A (including ISDR). The outcome of treatment was related to the core substitutions as well as to *IL28B *variation.

## Methods

### Samples

Pre-treatment serum samples from patients who were treated with peginterferon-alpha2a and ribavirin for chronic infection with hepatitis C virus genotype 1 in a previous study [18] were investigated. Fifty out of the 53 patients in the study were included in the present study. Two patients were excluded because serum samples were lacking, and one patient was excluded because sequencing revealed genotype 6e. The characteristics of the patients are shown in Table 1. The regional ethics committee in Gothenburg approved the study protocol, and a written informed consent was obtained from each patient.

**Table 1:** Virological and clinical characteristics of patients with hepatitis C virus infection

					Core amino acid			
							
Patient no	Genotype	Viral load (10^6 ^IU/ml)	Sex	Age (years)	70	91	*rs12979860*	End of treatment **response **^***a***^
R1	1a	4.36	M	52.6	R	C	CC	SVR
R2	1a	6.37	M	34.9	R	C	CC	SVR
R3	1a	7.84	M	45.8	R	C	CC	SVR
R4	1a	7.77	F	42.3	R	C	CC	SVR
R5	1a	7.05	M	45.3	R	C	CC	SVR
R6	1a	7.19	F	45.5	R	C	CC	SVR
R7	1a	5.54	F	46.9	R	C	CC	SVR
R8	1a	5.46	M	29.1	R	C	CT	SVR
R9	1a	6.18	M	50.7	R	C	CC	SVR
R10	1a	6.42	M	59.9	R	C	CC	SVR
R11	1a	5.85	M	46.4	R	C	CC	SVR
R12	1a	7.25	M	36.4	R	C	CT	SVR
R13	1a	6.43	M	57.5	R	C	CC	SVR
R14	1a	6.06	M	39.2	R	C	CT	SVR
R15	1a	6.63	F	47.1	R	C	CT	SVR
R21	1a	5.36	F	29.6	R	C	CT	SVR
R22	1a	5.55	F	28.7	R	C	CT	SVR
R23	1a	6.43	F	41.2	R	C	CC	SVR
R24	1a	6.10	M	51.3	R	C	CC	SVR
R25	1a	7.49	F	55.7	R	C	CC	SVR
R28	1a	7.79	M	41.5	R	C	CT	SVR
N1	1a	6.28	M	40.5	R	C	CT	non-SVR
N2	1a	6.25	M	50.3	R	C	CT	non-SVR
N3	1a	6.10	M	55.9	R	C	TT	non-SVR
N4	1a	7.05	M	47.9	R	C	TT	non-SVR
N5	1a	5.89	M	50.8	R	C	CT	non-SVR
N6	1a	6.42	F	48.1	P	C	CT	non-SVR
N7	1a	6.72	M	48.9	R	C	TT	non-SVR
N8	1a	7.35	M	54.6	R	C	TT	non-SVR
N9	1a	6.13	F	57.8	R	C	TT	non-SVR
N10	1a	6.72	M	54.6	R	C	TT	non-SVR
N11	1a	6.42	M	48.0	R	C	CT	non-SVR
N12	1a	7.32	F	48.4	R	C	CT	non-SVR
N13	1a	6.09	M	24.3	R	C	TT	non-SVR
N14	1a	6.31	M	35.0	R	C	CT	non-SVR
N20	1a	6.73	F	35.0	R	C	CC	non-SVR
N21	1a	7.15	F	45.0	R	C	CC	non-SVR
R16	1b	4.13	F	46.5	R	M	CC	SVR
R17	1b	4.94	M	31.5	R	M	CC	SVR
R18	1b	5.40	F	58.7	R	M	CT	SVR
R19	1b	6.23	F	38.4	R	L	CT	SVR
R20	1b	7.39	F	47.8	R	M	CT	SVR
R26	1b	7.17	M	46.6	R	L	CT	SVR
R27	1b	6.81	M	56.8	R	M	CT	SVR
R29	1b	7.55	M	57.0	Q	M	CT	SVR
N15	1b	6.08	F	56.5	Q	M	CT	non-SVR
N16	1b	6.57	F	58.5	Q	M	TT	non-SVR
N17	1b	7.37	M	48.9	Q	L	CT	non-SVR
N18	1b	6.69	M	62.8	Q	L	CT	non-SVR
N19	1b	6.70	F	54.2	Q	M	CT	non-SVR

^*a *^SVR, sustained virologic response; non-SVR, no sustained virologic response

#### HCV genotyping and sequencing

The genotype 1 subtype was assessed by phylogenetic analysis of the core region. The HCV-1b classification (13 patients) was further supported by sequencing of NS5A region. For sequencing, nucleic acid extraction was performed on 200 µl of patient serum using a MagNA Pure LC Instrument (Roche Applied Science, Mannheim, Germany). Complementary DNA was created with random primers. The core region (nucleotide, nt, 340 - 682) was subjected to nested PCR using the following primers: outer forward AAGGCCTTGTGGTACTGCCTG (274F), outer reverse ATGTACCCCATGAGGTCGGC (oka186R), inner forward GGAGGTCTCGTAGACCGTGCA (318F) and inner reverse GACCTTACCCAARTTMCGCGACCTA (709R).

Strains of genotype 1b were also amplified using primers outer forward GTCACAACTCCCATGCGAGC (6843F) and outer reverse AGCTCCGCCAAGGCAGAA (7412R), and in a second round using inner forward TTCCATGCTCACCGACCC (6887F) and inner reverse AATGGGCACCCGTGTACCAC (7319R), in order to sequence the ISDR (nt 6905-7299). Cycle sequencing was performed with 318F and 709R for the core region and with 6887F and 7319R for the ISDR. The sequences were read by an ABI 3100 Avant instrument (Applied Biosystems). The Sequencher (Gene Codes Corporation) and MacVector (MacVector Inc., Cambridge, UK) software were used for editing and phylogenetic analysis. The genotype of the sequences was established by comparison with GenBank sequences representative for all HCV genotypes.

#### Single Nucleotide Polymorphisms

Alleles of *rs12979860 *were assessed by first running a two-step PCR (15 s at 95°C; 60 s at 60°C) on an ABI 7300 instrument using primers rs12979860_F, GTGCCTGTCGTGTACTGAACCA and rs12979860_R, AGCGCGGAGTGCAATTCA and the Taqman MGB-probes rs12979860-C_P, FAM-CCTGGTTCGCGCCTT-MGB and rs12979860-T_P, VIC-CCTGGTTCACGCCT-MGB (SNP position underlined). Allelic discrimination was obtained by post-PCR read of fluorescence intensity from each of the fluorophores.

#### Statistical analysis

Statistical significance was evaluated by Fisher's exact test for group correlations, Mann-Whitney U test, Kruskal-Wallis test, student's t-test, logistic or linear regression analysis, as appropriate, using the SPSS software package (version 18, SPSS Inc, Chicago, Illinois).

#### Nucleotide sequence accession numbers

The nucleotide sequences will be available in GenBank with the accession numbers HQ729711 to HQ729773.

### Results

#### Baseline characteristics

Table 2 shows the baseline status of patients achieving and not achieving SVR. The mean age of all patients was 46.7 years. There was no significant difference in age of patients achieving SVR compared to those who did not (45.2 vs. 48.8 years, p = 0.09). Baseline HCV RNA below 5.6 log IU/mL was significantly associated with SVR. Neither subgenotype (1a vs. 1b) nor gender was associated with SVR.

**Table 2:** Host and viral baseline parameters in patients with and without treatment response

	SVR n = 29	non-SVR n = 21	Univariate p value
Age (mean)	45.2	48.8	0.09^a^
Number of patients < 45 / > 45 yrs	11 / 18	4 / 17	0.21^b^
Gender (m/f)	17 / 12	13 / 8	1.0^b^
Baseline HCV RNA (mean log IU/mL)	6.37	6.59	0.56^a^
Number with < 5.6 / > 5.6 log IU/mL	8 / 21	0 / 21	0.01^b^
Genotype 1a/1b	21 / 8	16 / 5	1.0^b^
Fibrosis (F0/F1/F2/F3/F4)^c^	0 / 10 / 13 / 4 / 0	2 / 4 / 4 / 7 / 2	0.19^d^
Core aa 70	28 R / 1 Q	15 R / 5 Q & 1 P	0.03^b^
Core aa 91	21 C / 6 M / 2 L	16 C / 3 M / 2 L	0.82^e^
*rs12979860*	16 CC / 13 CT / 0 TT	2 CC / 11 CT / 8 TT	0.0001^e^

^a ^Mann-Whitney U test.

^b ^Fisher's exact test.

^c ^Fibrosis was scored according to Ludwig and Batts, and was available for 34 patients.

^d ^Logistic regression.

^e ^Chi square test.

#### Subgenotypes, core mutations and treatment response

The virologic response was not associated with substitutions at residue 91. However, a poor response was associated with substitutions of core residue 70: One of the 7 patients (14%) with substitutions at residue 70 (six subtype 1b strains with Q70 and one subtype 1a strain with P70) achieved SVR, as compared with 28 of 43 patients (65%) carrying strains with R70 (p = 0.03).

Substitutions of core residues 70 and 91 were closely linked to subgenotype 1b: Six (5 non-SVR) of 13 genotype 1b strains had Q70, while only one of 37 subgenotype 1a strain had a substitution (P70) at this site (p = 0.0007). Similarly, all 1a strains had cysteine at residue 91, while in 1b 9 had methionine and 4 had leucine (p < 0.0001). This association between core variability and genotype was further explored by analysis of 3313 sequences from the HCV Database Project (http://hcv.lanl.gov/), showing a predominance (≥93%) of R70 in genotypes 1a, 2, 3 and 4, and high rates of Q70 in 1b, 5 and 6 (Table 3). In the 13 patients carrying subtype 1b strains, the correlation was strong: 7 of 8 responders had arginine (R70) and 5 non-responders had glutamine (Q70) at residue 70 (p = 0.005). In contrast, in the 37 patients with 1a infection, all the 21 patients with SVR carried HCV with R70, while 15 of the 16 non-SVR carried strains with R70.

**Table 3:** Distribution of amino acids at residue 70 and 91 of the core region

	Amino acid 70				Amino acid 91			
			
Genotype	Q	R	P	H	C	M	L	Total
1a	2%	98%	-	-	100%	-	-	920
1b	60%	35%	-	4%	1%	71%	28%	2022
2	-	100%	-	-	39%	4%	58%	83
3	-	93%	6%	-	99%	-	-	204
4	5%	95%	-	-	100%	-	-	19
5	86%	14%	-	-	-	-	100%	14
6	60%	13%	13%	15%	100%	-	-	55

A total of 3317 sequences found on 31^st ^of March 2010 in the The Hepatitis C Virus (HCV) Database Project (http://hcv.lanl.gov/) were analysed. Values less than 1% not shown.

#### Phylogenetic analysis of NS5A and ISDR substitutions

In order to find out if the variability at positions 70 and 91 was linked to subgroups of HCV-1b phylogenetic analysis of NS5A was performed, including the 13 strains from the present study as well as database sequences. The different core 70/91 variants were found in many sub-branches of the tree without clustering, indicating that they are not the result of a few historical mutations but evolve continuously (data not shown).

One substitution within ISDR (as compared with the HCV-J reference sequence) was observed in 9 of the 13 subgenotype 1b strains (7 H2218R, 2 H2218N), and 2 substitutions (H2219Y and D2225E) were seen in one strain. There was no association between ISDR substitution and treatment response.

#### IL28B SNP

**Figure 1:**
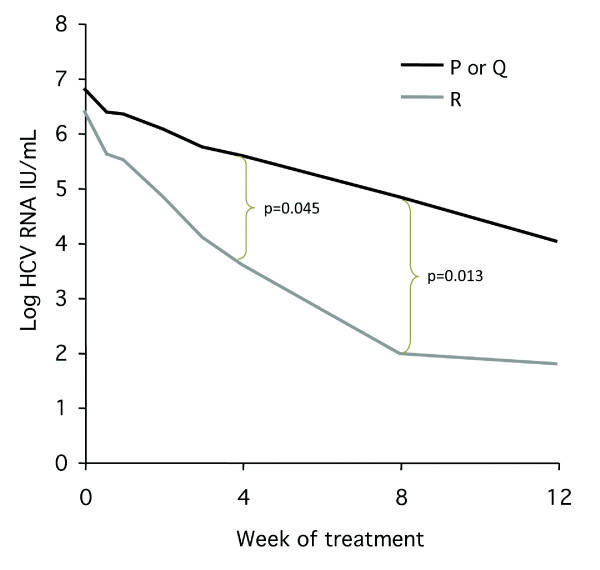
**HCV RNA declines during treatment in 24 patients carrying the rs12979860 CT genotype (heterozygotes) and HCV strains without (R, arginine) or with (P, proline or Q, glutamine) substitution at core residue 70**.

The *rs12979860 *T allele frequency was 40% as compared with 46% in 163 health subjects without HCV infection. The CC genotype, which has been associated with better treatment response in several previous studies, was identified in 18 patients, and 16 of them (89%) were achieved SVR. The unfavourable SNP genotype (TT) was found in 8 patients and none of them was responder. The remaining 24 patients were CT heterozygous, 13 (54%) of them achieved SVR. In an attempt to explore whether core variation might have an impact on response irrespective of *rs12979860*, the viral kinetics in patients carrying the CT SNP genotype and different HCV core variants was compared. As shown in Figure 1 the HCV RNA level after 4 and 8 weeks were significantly lower in the patients with CT_*rs12979860 *_carrying HCV with core R70 as compared with those with Q70 or P70. This impact also tended to influence the SVR rate which was higher (p = 0.06) in patients with R70 (67%; 12/18) than in those with Q70 and P70 (17%; 1/6).

### Discussion

Substitutions of core amino acids 70 and 91 have been associated with outcome of peginterferon/ribavirin treatment for chronic hepatitis C in Japanese patients infected with subgenotype 1b [11,19,20]. The result from the present study indicates that core variability may influence the treatment response also in Caucasian patients in Europe. However, only variation at residue 70 (but not residue 91) was associated with treatment response, and the impact was restricted to subgenotype 1b: Out of 13 patients with subgenotype 1b all the 5 non-SVR patients had Q70, while 7 of the 8 with SVR had R70. In contrast, 36 out of 37 patients (21 SVR, 16 non-SVR) with subgenotype 1a infection carried strains with R70. A similar lack of impact by core mutations in subgenotype 1a was recently reported by others [21].

The mechanism for the potential impact on treatment response is unknown, and it is possible that the variation at residue 70 is only indirectly linked to response. This seems to be supported by the fact that the typical core 70 amino acid differs between genotypes without any observed impact on response rates. Thus, the R70 variant, which has been associated with SVR in patients carrying HCV-1b strains, was observed in 98% of 1a sequences from GenBank (Table 3). Conversely, Q70, which seems to be associated with non-SVR in patients carrying subgenotype HCV-1b, is the predominant variant in genotypes 5 and 6, despite the fact that patients carrying these genotypes respond as well or better than patients infected with genotype 1a [22]. One study did not find any association of specific core amino acids in genotype 6f [23], and possibly the association of the C70 mutation to SVR is restricted to genotype 1b. These observations do not exclude a direct effect of core residue 70 on treatment response, because glutamine at position 70 might influence conformation and function of the core protein differently in HCV-1b than in HCV-1a or other genotypes. Indeed, a previous study has described that subgenotype 1a and 1b show different patterns of associations between genetic diversity and response to treatment [21]. Possibly, such differences may contribute to explain the reportedly higher SVR rate in patients with 1b than in those with 1a [24].

In order to find out if the core 70 or 91 variants might be linked to the phylogeny HCV-1b, core sequences from the 9 patients included in this study and 100 randomly chosen database sequences were compared by phylogenetic tree analysis. No clustering with respect to aa setup at aa 70 or 91 was observed, indicating that the evolution of Q70 is a sporadic but rather frequent event in HCV-1b.

Several previous reports from Japan have shown that substitutions in the ISDR in the NS5A region are associated with treatment response in patients infected with HCV-1b. In this study we found no impact of ISDR variability on response in patients carrying HCV-1b, but considering that only 13 patients were investigated, such an association might be missed.

The main focus of the present study was not the importance of the recently identified single nucleotide polymorphisms (SNP) upstream the *IL28B *gene. However, because this variability might influence the interpretation of core variability, the *rs12979860 *SNP was analysed. The results show that this SNP has a strong impact on treatment response, as 16 out of 18 patients with CC as opposed to no patients with TT at *rs12979860 *achieved SVR. This strong impact of *IL28B *variability might bias the effect of HCV-core variability on SVR, or the strong host factor may overshadow an effect of the viral factor. The risk for such an effect is probably lower in Japan where most patients carry the favourable CC genotype at *rs12979860*, than in Caucasian populations where also the CT and TT *IL28B *genotypes are frequent. However, we did not find any significant association between *rs12979860 *and core aa 70 and an impact of core variation at residue 70 on response was observed also within the group that carried CT at *rs12979860*: the decline in HCV RNA was steeper in patients infected with HCV with R70 as compared with Q70. This finding indicates that the effect of core variability is not solely dependent of *IL28B*, and that HCV core analysis might be clinically relevant, primarily in patients with the CT_*rs12979860 *_genotype. Multivariate analysis was not performed, as the number of samples in the study is low, with the risk of generating false positive results in the subgroups.

### Conclusions

In conclusion, the results support that substitutions at position 70 but not at position 91 in the core protein of HCV-1b are associated with treatment response in Caucasian patients with chronic hepatitis C. The observation needs to be confirmed in studies with larger number of patients with HCV-1b infection taking ethnicity and IL28B polymorphisms into consideration.

### Competing interests

The authors declare that they have no competing interests.

### Authors' contributions

EA and MLI designed the study, contributed to acquisition, analysis and interpretation of data and were responsible for drafting the manuscript. BA, AE, ML, SN, GN, TW, RW, JW made substantial contributions to the acquisition of data, critically revised the manuscript and gave approval of the final version.

## Pre-publication history

The pre-publication history for this paper can be accessed here:

http://www.biomedcentral.com/1471-2334/11/124/prepub
